# Global cognitive functioning in TRD and non-TRD patients: longitudinal real-world evidence from the PROMPT study

**DOI:** 10.1192/j.eurpsy.2026.11389

**Published:** 2026-07-31

**Authors:** V. T. H. Wahner, S. Jörgens, B. T. Baune, PROMPT consortium

**Affiliations:** 1 Department of Psychiatry, University of Münster, Münster; 2 Department Hamm 2, Hochschule Hamm-Lippstadt, Hamm, Germany; 3 Department of Psychiatry, University of Melbourne; 4 Florey Institute of Neuroscience and Mental Health, Parkville, Australia

## Abstract

**Introduction:**

The exact role of cognitive functioning (CF) and impairment in treatment-resistant depression (TRD) is not well understood. In particular, the lack of longitudinal and comparative data with non-TRD patients represents a major gap in the current literature.

**Objectives:**

This study sought to compare 12-week trajectories of global CF between TRD and non-TRD patients in a real-world setting.

**Methods:**

This prospective observational study included 146 inpatients with major depressive disorder (MDD) who were assessed with a standardized cognitive test battery (BAC-A) and structured clinical interviews at baseline, week 8, and week 12. Fifty-four patients (mean age = 48.5±15.5; 48% female) met the criteria for TRD, defined as nonresponse to at least two antidepressant regimens during the current episode, and were compared with ninety-two non-TRD patients (mean age = 33.9±12.8; 50% female). Composite cognitive *z*-scores (age- and sex-adjusted) were analysed using linear mixed-effects models to examine the effects of group, time and their interaction. In a second step, the model was adjusted for symptom severity (MADRS scores), recurrence, years of education, current medication (benzodiazepines, antipsychotics) and psychiatric comorbidities (anxiety disorders, personality disorders).

**Results:**

Global CF improved significantly over 12 weeks (β = 0.29, *p* < .001). The TRD group showed lower baseline CF (β = -0.45, *p* = .032). This group effect was no longer significant after adjustment for clinical covariates. Higher education predicted better CF (β = 0.12, *p* < .001), whereas antipsychotic use was associated with poorer CF (β = -0.74, *p* < .001). The non-significant group × time interaction indicated similar trajectories between groups. Random-effects variances (intercept = 1.05; slope = 0.00) suggested substantial between-subject variability at baseline and parallel improvement trajectories. Figure 1 depicts group mean trajectories of global CF over 12 weeks.

**Image 1:**
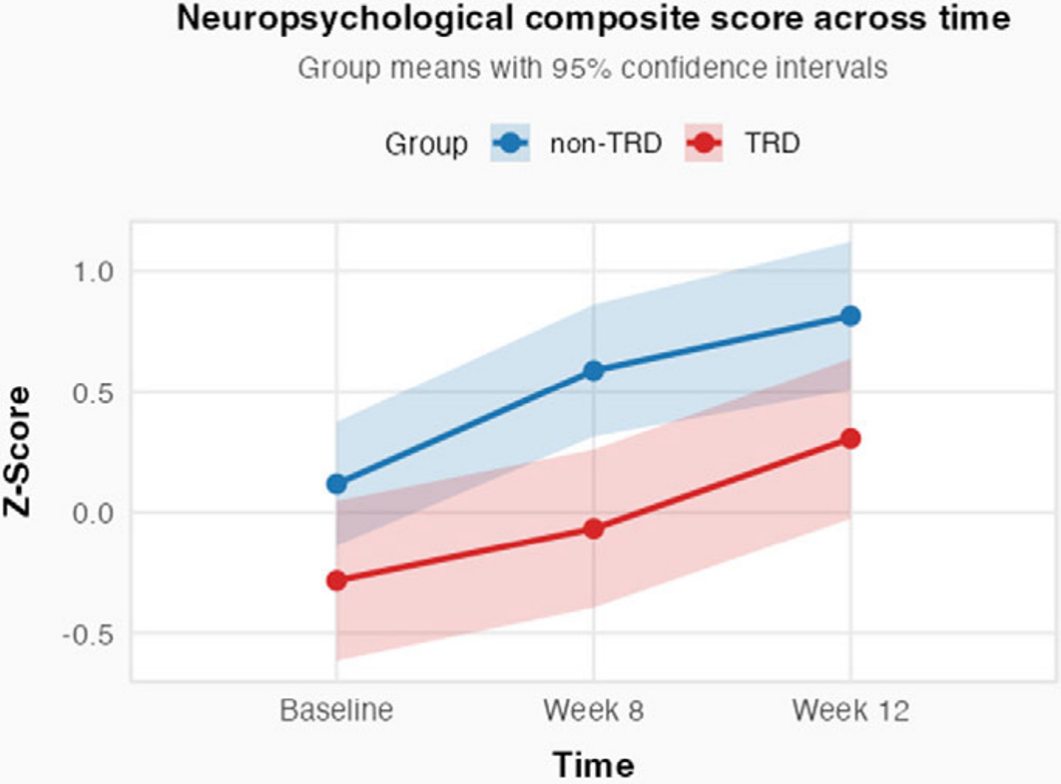
Image 1: Long description.

**Conclusions:**

Observed differences in global CF between TRD and non-TRD patients appear to reflect underlying clinical characteristics rather than treatment resistance per se. Future studies should investigate group differences at the domain level and account for substantial between-subject variability in their analyses.

**Disclosure of Interest:**

V. Wahner: None Declared, S. Jörgens: None Declared, B. Baune Consultant of: BTB received speaker/consultation fees from: AstraZeneca, Lundbeck, Pfizer, Takeda, Servier, Bristol Myers Squibb, Otsuka, LivaNova, Boehringer-Ingelheim, Biogen and Janssen-Cilag.

## Long descriptions

### Image 1: Long description

The X-axis represents Time with three intervals: Baseline, Week 8, and Week 12. The Y-axis represents Z-Score ranging from -0.5 to 1.0. Two groups are plotted with 95 percent confidence intervals shown as shaded bands.

* The non-T R D group (blue line) starts at a Z-score of approximately 0.1 at Baseline, increases to 0.6 at Week 8, and reaches approximately 0.8 at Week 12. The blue shaded confidence interval remains entirely above the 0.0 line after Baseline.

* The T R D group (red line) starts at a lower Z-score of approximately -0.3 at Baseline, rises slightly to just below 0.0 at Week 8, and reaches approximately 0.3 at Week 12. The red shaded confidence interval overlaps with the 0.0 line throughout the duration.

Both groups show a positive linear trend over time, but the non-T R D group maintains higher composite scores at every time point compared to the T R D group.

Navigate back to Image 1:.

